# “Randomized trial of physical activity on quality of life and lung cancer biomarkers in patients with advanced stage lung cancer: a pilot study”

**DOI:** 10.1186/s12885-021-08084-0

**Published:** 2021-04-01

**Authors:** Brett C. Bade, Geliang Gan, Fangyong Li, Lingeng Lu, Lynn Tanoue, Gerard A. Silvestri, Melinda L. Irwin

**Affiliations:** 1 Department of Internal Medicine, Section of Pulmonary, Critical Care, and Sleep Medicine, Yale University School of Medicine, P.O. Box 208057 300 Cedar Street TAC – 441 South, New Haven, CT 06520-8057 USA; 2 Yale Center for Analytical Sciences, Yale School of Public Health, New Haven, USA; 3 Department of Chronic Disease Epidemiology, Yale University School of Public Health, New Haven, USA; 4 Division of Pulmonary, Critical Care, and Sleep Medicine, Department of Internal Medicine, Medical University of South Carolina, New Haven, USA

**Keywords:** Physical activity, Exercise, Lung Cancer, Biomarkers, Mobile health, Quality of life

## Abstract

**Background:**

Lung cancer survivors need more options to improve quality of life (QoL). It is unclear to what extent patients with advanced stage disease are willing to participate in home-based physical activity (PA) and if these interventions improve QoL. The goal of our study was to determine interest in participating in our 3-month home-based walking regimen in patients with advanced stage lung cancer. We used a randomized design to evaluate for potential benefit in PA and patient-reported outcomes.

**Methods:**

We performed an open-label, 1:1 randomized trial in 40 patients with stage III/IV non-small cell lung cancer (NSCLC) evaluating enrollment rate, PA, QoL, dyspnea, depression, and biomarkers. Compared to usual care (UC), the intervention group (IG) received an accelerometer, in-person teaching session, and gain-framed text messages for 12 weeks.

**Results:**

We enrolled 56% (40/71) of eligible patients. Participants were on average 65 years and enrolled 1.9 years from diagnosis. Most patients were women (75%), and receiving treatment (85%) for stage IV (73%) adenocarcinoma (83%). A minority of patients were employed part-time or full time (38%). Both groups reported low baseline PA (IG mean 37 (Standard deviation (SD) 46) vs UC 59 (SD 56) minutes/week; *p* = 0.25). The IG increased PA more than UC (mean change IG + 123 (SD 212) vs UC + 35 (SD 103) minutes/week; *p* = 0.051)). Step count in the IG was not statistically different between baseline (4707 step/day), week 6 (5605; *p* = 0.16), and week 12 (4606 steps/day; *p* = 0.87). The intervention improved EORTC role functioning domain (17 points; *p* = 0.022) with borderline improvement in dyspnea (− 13 points; *p* = 0.051) compared to UC. In patients with two blood samples (25%), we observed a significant increase in soluble PD-1 (219.8 (SD 54.5) pg/mL; *p* < 0.001).

**Conclusions:**

Our pilot trial using a 3-month, home-based, mobile health intervention enrolled over half of eligible patients with stage III and IV NSCLC. The intervention increased PA, and may improve several aspects of QoL. We also identified potential biomarker changes relevant to lung cancer biology. Future research should use a larger sample to examine the effect of exercise on cancer biomarkers, which may mediate the association between PA and QoL.

**Clinical trial registration:**

Clinicaltrials.gov (NCT03352245).

## Background

With continued progress in tobacco smoking cessation, lung cancer screening, and systemic treatment options, lung cancer survival is improving, [1] and long-term lung cancer survivors (i.e., > 5 years) are emerging [2]. Though impaired quality of life (QoL), high symptom burden, [2] functional impairment, [3] and depression [4] are common in lung cancer and associated with worsened outcomes, [5] treatments directed at these impairments are disappointingly infrequent [6]. Thus, there is a continued need for interventions to improve QoL and minimize side effects associated with the diagnosis and treatment of lung cancer.

Physical activity (PA) and exercise tolerance are associated with a lower risk of lung cancer mortality as well as longer overall survival and higher QoL. In studies with predominantly non-small cell lung cancer (NSCLC) patients, 6-min walk test, [7] peak oxygen uptake (or consumption), [8] daily step count, [9] and self-reported PA (including any activity that increases bodily movement) [10, 11] are all associated with survival. Compared to patients with the lowest exercise tolerance, Jones and colleagues reported a hazard ratio for all-cause mortality of 0.56–0.64 [8]. Observational studies have similarly reported improvements in PA to be associated with better QoL [12, 13]. Since several types of PA appear beneficial and the “optimal” PA recommendations for patients are not yet clear, guidelines recommend that lung cancer survivors maintain a physically active lifestyle to improve symptoms, physical function, and QoL [14, 15]. Unfortunately, most patients with lung cancer are inactive [16]. Despite the existing data and guideline recommendations, PA is rarely discussed with lung cancer survivors, and there is no standard-of-care intervention [17].

Patients with advanced NSCLC (i.e., stages III/IV) are in the most vulnerable position, as they often have the severest symptoms, QoL, and impairments in physical function [18–20]. Yet, activity interventions are infrequently studied in patients with advanced disease [21, 22]. Counterintuitively, patients with more significant baseline impairments may obtain the most benefit from PA interventions [23, 24]. Since Jensen and colleagues showed that physical activity or physical therapy was feasible and potentially beneficial in > 90% of cancer patients in a palliative care inpatient ward, [25] physicians should look for opportunities to implement physical activity safely. Low impact PA (i.e., walking) is recommended for many patients. A recent walking intervention showed improvement in anxiety and depression symptoms in patients with predominately early stage lung cancer, [26]. By contrast, a similarly-sized PA trial in patients with advanced stage lung cancer (utilizing supervised exercise sessions) did not show improvement in QoL [27]. We hypothesize that PA may improve QoL in patients with advanced stage lung cancer, and patients with metastatic lung cancer may be more willing and able to participate in home-based interventions facilitated by mobile health (mHealth). Our prior work has shown that home-based PA monitoring with accelerometers is feasible in this population, [28] and a combined intervention (including a teaching session, individualized walking goals, and gain-framed text messages) had high patient satisfaction and increased subjects’ PA [29]. In this study we sought to determine interest in participating in a home-based PA regimen in patients with stage III-IV NSCLC. We used a randomized design to examine the effects of our intervention on PA;dyspnea, QoL, and depression scores; and biomarkers.

## Methods

### Study setting and participants

We performed an open-label, pilot, randomized, controlled trial in 40 patients with stage III or IV NSCLC. Our protocol was approved by Yale Cancer Center’s Thoracic Oncology Disease Aligned Research Team (DART) and Yale University’s institutional review board/Human Investigations Committee (HIC# 2000022225). As a prospective intervention, the study was registered with clinicaltrials.gov (NCT03352245; first posted 24/11/2017). As a pilot study, size was chosen based on feasibility of recruitment in 1 year. Using Aaronson and colleagues’ work on the EORTC, [30, 31] *n* = 40 provides 80% power to detect an improvement of 20.4 points in Global QOL in intervention group at 12 weeks (relative to control arm) with a significance level of 0.05 using a two-sided two sample t test.

We enrolled patients for the study between 10/22/2018 and 1/22/2020. Patients were screened from our Thoracic Oncology Program clinic, which includes medical oncology, radiation oncology, pulmonology, and thoracic surgery disciplines. Inclusion criteria were: pathologic evidence of stage III or IV NSCLC (any stage of treatment), approval of the treating clinician, access to a smartphone, willingness to wear a wrist-bound accelerometer for 3 months, willingness to receive twice daily text messages, and baseline physical inactivity (i.e, < 150 min/week of moderate-intensity physical activity, < 75 min/week of vigorous-intensity physical activity, or a combination). Intensity of PA was determined by the patient and gauged via the “talk test,” with the ability to talk during moderate-intensity exercise. During vigorous-intensity exercise, only a few words are able to be spoken before having to pause for a breath [32]. Due to slower than anticipated initial enrollment, we expanded our criteria to potentially include patients with early stage lung cancer. Due to acceleration in enrollment, the expanded enrollment criteria were not utilized, and we completed our analysis as originally planned. Exclusion criteria were inability to safely walk, memory impairment, and communication barriers. Patients who were not expected to survive beyond the intervention period (3 months) were not enrolled.

### Study procedures

#### Recruitment

**Fig. 1:**
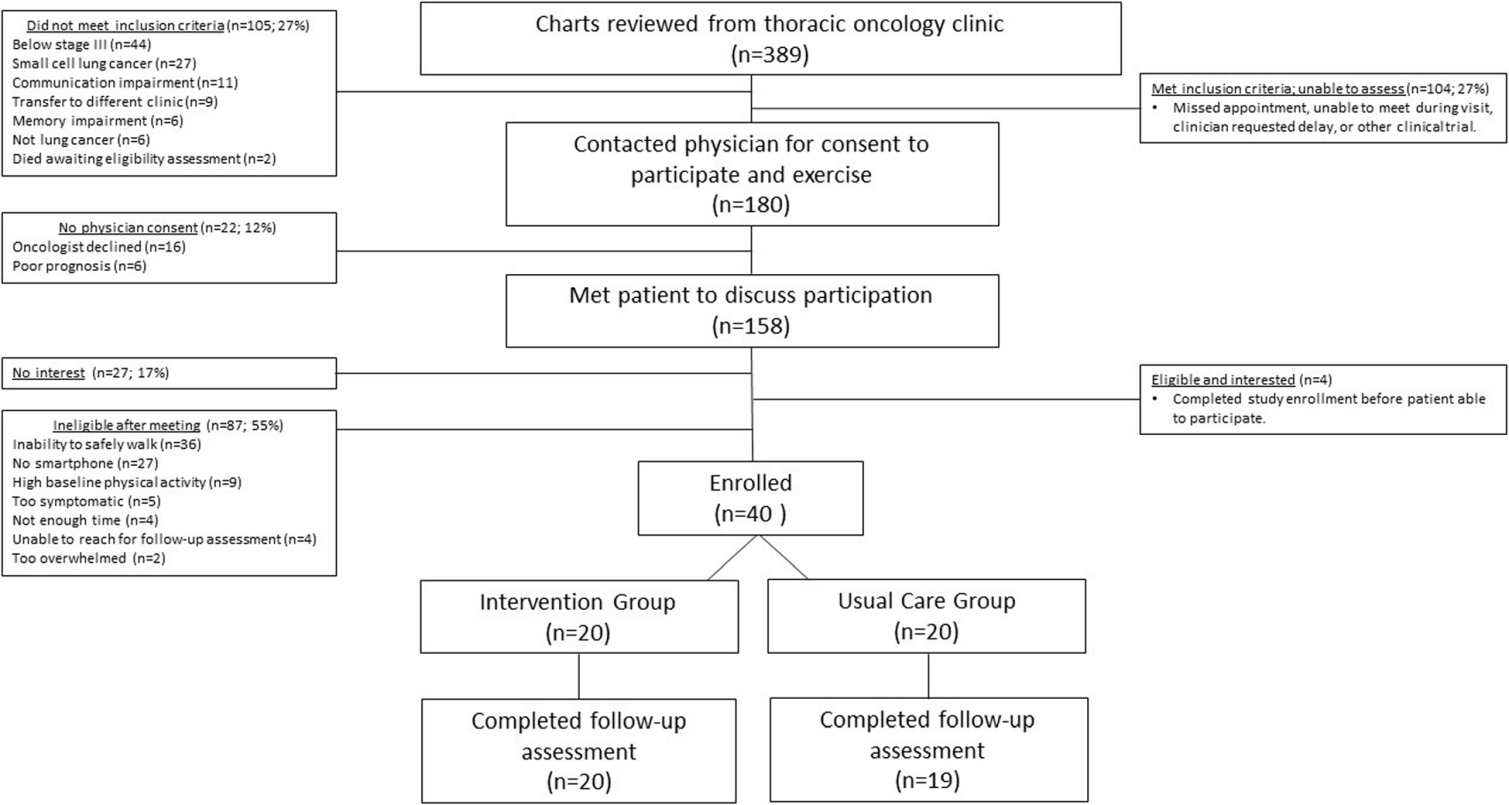
Consort Diagram

Patients were screened via the medical record prior to their clinic visit. Patients meeting inclusion criteria were reviewed with the treating oncologist. Study staff met eligible patients during a clinic visit to discuss study participation. If physician consent was provided and patients were willing to participate, we provided our consent document for the patient to review, and a separate in-person visit was arranged for enrollment (Fig. 1). At the enrollment visit, risks/benefits of study participation were re-discussed, patients were given the opportunity to ask questions, and consent was obtained.

#### Baseline and follow-up symptom, QoL, depression, and PA measures

Patients completed paper questionnaires including the modified Medical Research Council Dyspnea scale (MMRC), the Modifiable Activity Questionnaire, [33] European Organisation for the Research and Treatment of Cancer Quality of Life Core Questionnaire (EORTC-QLQ-C30), and the Patient Health Questionnaire-9 depression scale (PHQ-9). The MMRC dyspnea scale is generally used for patients with chronic lung disease and scored 0–4 (higher score indicating more dyspnea) [34]. The Modifiable Activity Questionnaire lists weekly physical activities and time spent to obtain the total weekly time spent in moderate- and vigorous PA. Approval for EORTC-QLQ-C30 use for research was obtained prior to study initiation. The EORTC-QLQ-C30 is a 30-item questionnaire that has been used extensively in multiple types of cancer. The questionnaire is scored in values 0–100 and provides a global health status/QoL score (higher numbers indicating better quality of life), functional scales (physical, role, emotional, cognitive, and social functioning; higher score indicating better functioning), and symptom scales (fatigue, nausea/vomiting, pain, dyspnea, insomnia, appetite loss, constipation, diarrhea, and financial difficulties; higher score indicating higher symptom distress). We also calculated the QLQ-C30 Summary Score, that incorporates most items (excepting financial impact and global health status) and is more sensitive to changes in QoL for patients with NSCLC [35]. For the EORTC-QLQ-C30, “mildly” and “moderately” clinically meaningful changes have been estimated at > 5 and > 10 points, respectively [31, 36]. The PHQ-9 is a self-administered, 9-item questionnaire scored 0–27, with higher score indicating higher likelihood and severity of depression [37].

#### Biomarkers

A fasting blood sample was collected at baseline (prior to week 1) and 3-months. Metabolic, inflammatory, and lung cancer-specific biomarkers were chosen based on their likelihood to (1) be influenced by PA and (2) predict cancer outcomes. Prior work in breast cancer has shown that inflammatory biomarkers are inversely related to PA at baseline, metabolic biomarkers vary inversely with PA, and metabolic biomarkers are associated with increased risk of death [38–40]. Biomarkers were analyzed following the manufacturer’s instructions (Life Technologies Co., Carlsbad, CA): insulin (Cat. No. KAQ1251, Lot No. 2001–6125), leptin (Cat. No. KAC2281, Lot No. 1910–5427), C-reactive protein (CRP) (Cat. No. KHA0031, Lot No. 226513–011), soluble programmed cell death protein 1 (sPD-1) (Cat. No. BMS2214, Lot No. 227479–004), and soluble programmed cell death ligand-1 (sPD-L1) (Cat. No. BMS2212, Lot No. 219915–006). Each sample was run in duplicate for each marker, and the average of the concentrations for each marker was calculated for each patient.

#### Randomization and intervention

Using an envelope system based on timing of enrollment, patients were randomly assigned to either Intervention or Usual Care (UC) groups with a 1:1 ratio. The Intervention Group (IG) received (1) a 15 min in-person teaching session regarding the benefits of physical activity in lung cancer; (2) a FitBit® (San Francisco, CA, United States) Flex2 accelerometer (and set-up on their phone); (3) individualized walking goals based on their average daily step count during week 1; and (4) twice daily gain-framed text messages. Gain-framed messages prioritize the benefits of a behavior and have been used in smoking cessation [41] and PA implementation [42]. The process and mechanism of text messaging were reviewed with our institution’s information technology department and Chief Privacy Officer. The messages were delivered via a Health Information Portability and Accountability Act (HIPAA) compliant program.

Our intervention has been previously described [29]. In brief, step counts were recorded by the study team daily. Subjects were asked to wear the accelerometer always (excepting during device charging), try to keep the accelerometer dry, and to maintain their “normal” activity level for 1 week following enrollment to establish a step count baseline. In subsequent weeks, we recommended increasing steps by 400/day above their average daily step count. The 400 steps/day increase is based on prior work in patients with chronic obstructive pulmonary disease [43] and was used in our pilot intervention [29]. As an example, if the average daily step count after week 1 was 4000 steps, we recommended 4400 steps/day for week 2. In subsequent weeks, additional increases by 400 steps/day were recommended if patients met the recommended goal. If the goal was not met, the prior week’s recommendation was maintained. If patients achieved an average of 10,000 steps/day, they were recommended to maintain their current PA. Each patient in the IG received the same text messages; personalization was provided by the patient’s first name, their individual step count, and their current step count goal. If missing step counts for one day were noted, the study team replaced a scheduled text message with one requesting a call to the research team. If step counts were missing for > 2 days, patients were called by the study team to evaluate for adverse events or trouble-shoot technological issues. The UC group was thanked for their participation and advised to remain physically active as recommended by their clinical team.

#### Follow-up visits

At the end of 12 weeks, a follow-up visit was arranged, during which questionnaires were repeated, a fasting blood draw was re-collected, adverse events were queried, a feedback questionnaire (evaluating satisfaction and willingness to participate in follow-up studies) was completed, and a FitBit® Flex2 accelerometer was provided to the UC group. All subjects were allowed to keep their accelerometers. If necessary, questionnaire completion by phone was permitted (and included in the initial protocol).

Patients in both groups were asked to call or e-mail the study team if they were hospitalized, visited the emergency room (ER), fell, or developed other pain (especially chest pain) that they attributed to walking. In the event of (1) a serious adverse event or (2) the PHQ-9 indicated any level of suicidality, the treating oncologist and study primary investigator reviewed the case to ensure appropriate clinical treatment, evaluate potential causality, and review appropriateness of study continuation.

#### Statistical analyses

Patient demographics and clinical characteristics were summarized with frequency and percentage for categorical variables. Continuous variables were summarized with mean and standard deviation or median and range. Comparisons between groups used t test for continuous variables and Chi-square test for categorical variables Changes in QoL scores and biomarkers were analyzed via mixed effects repeated measures modeling, adjusting for sex. The baseline variables were constrained to be equal while linear contrasts were carried out to test the group difference. This analysis included all randomized subjects [44]. The relationship between baseline characteristics and physical activity in the intervention group was evaluated via a simple linear regression. Pearson correlation coefficients were utilized to determine the relationship between increases in physical activity and QoL scores. All statistical analyses were conducted using SAS 9.4 (Cary, NC). Significance level was set at *p* < 0.05, two-sided.

## Results

### Baseline characteristics

Over approximately 15 months, we reviewed the charts of 389 patients from the thoracic oncology clinic (Fig. 1). We screened 158 for interest and eligibility, with 56% (40/71) of those eligible being enrolled.

Study participants had an average age of 65 years and were enrolled an average of 1.9 years from their original lung cancer diagnosis (range 0.1–10.7 years; Table 1). Most patients were actively receiving treatment (85%; *p* = 0.66); immunotherapy (41%) and targeted therapy (29%) were the most common active treatments (*p* = 0.23). Most patients were women (75%), non-Hispanic white (85%), and completed education beyond high school (70%). A minority of patients were employed part-time or full time (38%). Adenocarcinoma was the most common histology (83%), and most patients had stage IV disease (73%). The Intervention and UC groups were generally well-matched, though the UC group was more likely to be female (IG 60% vs UC 90%; *p* = 0.03).

**Table 1:** Patient Demographics

	Intervention Group (***n*** = 20)	Usual Care Group (n = 20)	Total (***N*** = 40)	***P***-value
Age				0.23
Mean (SD^a^)	66.55 (7.28)	63.20 (9.80)	64.88 (8.69)	
Median (Range)	68.5 (55.0–79.0)	63.5 (42.0–78.0)	66.0 (42.0–79.0	
Duration Between Cancer Diagnosis and Enrollment (median, range, years)	1.6 (0.1–6.0)	2.2 (0.2–10.7)	1.9 (0.1–10.7)	0.14
Sex				**0.03**
Female	**12 (60%)**	**18 (90%)**	**30 (75%)**	
Male	**8 (40%)**	**2 (10%)**	**10 (25%)**	
Race				0.66
White	18 (90%)	16 (80%)	34 (85%)	
Other	2 (10%)	4 (20%)	6 (15%)	
Body Mass Index (mean, SD)	27.75 (8.44)	26.52 (5.73	27.13 (7.14)	0.59
Highest level of education completed				0.17
High School	8 (40%)	4 (20%)	12 (30%)	
College and above	12 (60%)	16 (80%)	28 (70%)	
Marital Status				0.74
Married or Living with Partner	13 (65%)	12 (60%)	25 (62.5%)	
Divorce, Widowed, Living Alone	7 (35%)	8 (40%)	15 (37.5%)	
Work Status				0.61
Employed full-time	7 (35%)	5 (25%)	12 (30%)	
Employed part-time	2 (10%)	1 (5%)	3 (7.5%)	
Other	11 (55%)	14 (70%)	25 (62.5%)	
Cancer Type				0.49
Adenocarcinoma	18 (90%)	15 (75%)	33 (82.5%)	
Squamous Cell Carcinoma	1 (5%)	2 (10%)	3 (7.5%)	
Other or Mixed Histology	1 (5%)	3 (15%)	4 (10%)	
Stage				0.77
IIIA	3 (15%)	2 (10%)	5 (12.5%)	
IIIB	2 (10%)	4 (20%)	6 (15%)	
IV	15 (75%)	14 (70%)	29 (72.5%)	
Performance Status				1.00
ECOG 0	3 (15%)	4 (20%)	7 (17.5%)	
ECOG 1	17 (85%)	16 (80%)	33 (82.5%)	
Tobacco Smoking History				0.69
Current Smoker	1 (5%)	0 (0%)	1 (2.5%)	
Former Smoker	16 (80%)	15 (75%)	31 (77.5%)	
Never Smoker	3 (15%)	5 (25%)	8 (20%)	
Cancer Treatment Status				0.23^b^
Receiving Treatment	16 (80%)	18 (90%)	34 (85%)	
Chemotherapy only	2	2	4	
Chemo-immunotherapy	5	1	6	
Immunotherapy only	6	8	14	
Targeted therapy	3	7	10	0.66^c^
Post-treatment	4 (20%)	2 (10%)	6 (15%)	

^a^ SD: Standard Deviation

^b^ *P*-value comparing distribution of current treatment between IG and UC groups

^c^ *P*-value comparing Receiving Treatment vs. Post-treatment

### Physical activity

We collected PA data via self-report in both groups and daily step counts (via the FitBit® Flex2) in the Intervention Group only (Table 2). As assessed via the PA questionnaire, at baseline patients reported an average of 48 min of moderate- to vigorous-intensity PA per week. At 3 months, the IG reported a greater increase in PA min/week: IG + 123 (SD 212) vs UC + 35 (SD 103) minutes; *p* = 0.051). Regarding step counts from the FitBit® in the IG, patients provided “usable” data (> 200 steps/day for > 5 of 7 days) for 90% of weeks during the study. Patients in the intervention group averaged 4707 steps/day at baseline (week 1), 5605 steps/day at the midpoint (week 6), and 4606 steps/day at last week (week 12). Among the IG, the mean change per patient (compared to baseline) was a non-significant increase of 893 steps/day at week 6 (*p* = 0.16) and a decrease of 107 steps/day from baseline at week 12 (*p* = 0.87). Individualized walking goals were only met in 21% of weeks.

**Table 2:** Physical Activity Baseline and Follow-up in Both Study Groups

	Intervention Group (n = 20)	Usual Care Group (n = 20)	Total (N = 40)	***P***-value
**Self-Reported Physical Activity (minutes)**				
**Baseline weekly exercise minutes (moderate + vigorous)**				
Mean (SD)	37.0 (46.4)	58.8 (55.8)	47.9 (51.8)	0.25
Median (Range)	8 (0–125)	60 (0–140)	30 (0–140)	
**Weekly exercise minutes (moderate + vigorous) at study completion**				
Mean (SD)	160.1 (231.2)	92.6 (124.4)	127.2 (187.7)	0.52
Median (Range)	43 (0–861)	0 (0–330)	15 (0–861)	
**Change between Week 1 and Week 12**				
Mean (SD)	123.01 (212.0)	34.7 (102.7)	80 (171.6)	0.051^a^
Median	8 (−60–811)	0 (− 120–225)	0 (−120–811)	
**Step Counts**				
Average step count, baseline, (range, *n* = 19) (week 1)	4707 (1568-12,222)	N/A	N/A	
Average step count, midway (range, *n* = 18) (week 6)	5605 (1079-9764)			0.16
Mean change (midway vs baseline, n = 18; range)	893 (− 5139–4874)			
Average step count, last week (range)	4606 (746–10,238)			0.87
Mean change (baseline vs last week; range)	−107 (− 6495-4163)			

^a^*p*-value is calculated in linear model controlling patient gender and baseline exercise minutes

### Quality of life

The baseline questionnaire scores by study group were summarized in Table 3. Patients in the UC group reported a significantly higher constipation score. In the entire cohort at baseline, the average QLQ summary and global health status scores were 85.08 (standard error (SE) 2.16) and 73.32 (SE 3.67), respectively (Table 4). At end of the study, both groups reported higher QLQ summary score (IG 87.36 (SE 2.64); UC 86.05 (SE 2.79)). The global health status score improved in the IG (76.48 (SE 4.35)) but worsened in the UC group (UC 68.69 (SE 4.77)). The between-group difference of score change was not significant for the QLQ summary score (1.31 (SE 3.02); *p* = 0.67) nor the global health status (7.79 (SE 5.51); *p* = 0.17).

**Table 3:** Baseline Patient-Reported Outcomes by Study Group

Score Type	Intervention Group	Usual Care	*P* value
**European Organisation for the Research and Treatment of Cancer (EORTC) Core Quality of life Questionnaire**			
**Overall and Functioning Domains**			
QLQ summary score	84.53 (12.45)	83.89 (11.15)	0.87
Global health status (SE)	70.42 (21.88)	77.08 (18.90)	0.31
Physical functioning	83.00 (16.96)	82.81 (14.46)	0.97
**Role functioning**	**79.17 (25.86)**	**91.67 (13.79)**	**0.07**
Emotional functioning	83.33 (15.53)	85.00 (15.67)	0.74
Cognitive functioning	91.67 (13.79)	85.83 (17.33)	0.25
Social functioning	81.67 (21.56)	84.17 (15.74)	0.68
**Symptom Domains**			
Fatigue	23.33 (19.04)	25.56 (20.10)	0.72
Nausea and vomiting	7.50 (13.76)	3.33 (10.26)	0.28
Pain	12.50 (19.40)	13.33 (26.27)	0.91
Dyspnea	**28.33 (27.09)**	**20.00 (16.75)**	**0.25**
Insomnia	18.33 (25.31)	25.00 (23.88)	0.4
Appetite loss	13.33 (22.69)	11.67 (22.36)	0.82
Constipation	**6.67 (13.68)**	**30.00 (32.26)**	**0.006**
Diarrhoea	10.00 (21.90)	11.67 (19.57)	0.8
Financial Difficulties	18.33 (29.57)	16.67 (22.94)	0.84
**MMRC Dyspnea Scale**	1.00 (1.05)	1.10 (0.85)	0.75
**PHQ-9 Total Score (omits Q10)**	4.00 (4.10)	3.26 (2.73)	0.52

**Table 4:** Overall Patient-Reported Outcomes by Study Group (Mixed Effects Model)

Score Type	Score at Week 1	Intervention Group		Usual Care		Difference of score change	***P***-value
		Score at Week 12	Score Change	Score at Week 12	Score Change		
**European Organisation for the Research and Treatment of Cancer (EORTC) Core Quality of life Questionnaire**							
**Overall and Functioning Domains**							
QLQ summary score	85.08 (2.16)	87.36 (2.64)	2.28 (2.18)	86.05 (2.79)	0.97 (2.22)	1.31 (3.02)	0.668
Global health status (SE)	73.32 (3.67)	76.48 (4.35)	3.16 (4.11)	68.69 (4.77)	−4.63 (4.20)	7.79 (5.51)	0.166
Physical functioning	84.02 (2.79)	88.13 (3.10)	4.11 (2.50)	88.76 (3.31)	4.74 (2.54)	−0.64 (3.41)	0.853
**Role functioning**	**85.51 (3.84)**	**93.60 (5.27)**	**8.09 (5.33)**	**76.55 (5.76)**	**−8.95 (5.45)**	**17.04 (7.16)**	**0.022**
Emotional functioning	85.11 (2.74)	87.79 (3.07)	2.68 (2.89)	88.22 (3.38)	3.11 (2.95)	−0.43 (3.83)	0.911
Cognitive functioning	90.93 (2.74)	91.35 (3.02)	0.41 (2.26)	93.40 (3.22)	2.47 (2.31)	−2.05 (3.13)	0.515
Social functioning	83.91 (3.41)	79.69 (5.51)	−4.22 (5.38)	88.31 (5.90)	4.40 (5.50)	−8.62 (7.45)	0.254
**Symptom Domains**							
Fatigue	22.46 (3.51)	18.04 (5.54)	−4.43 (4.85)	23.21 (5.79)	0.75 (4.97)	−5.18 (6.88)	0.456
Nausea and vomiting	6.32 (2.19)	3.88 (4.15)	−2.44 (4.18)	12.53 (4.42)	6.21 (4.28)	−8.65 (5.80)	0.144
Pain	12.21 (4.06)	13.88 (4.79)	1.67 (4.77)	13.47 (5.29)	1.26 (4.87)	0.41 (6.28)	0.948
Dyspnea	**23.31 (4.06)**	**17.12 (5.15)**	**−6.19 (4.88)**	**30.43 (5.60)**	**7.12 (4.99)**	**−13.31 (6.62)**	**0.051**
Insomnia	19.64 (4.40)	20.22 (6.47)	0.58 (6.37)	16.15 (6.99)	−3.49 (6.52)	4.07 (8.71)	0.643
Appetite loss	12.24 (3.92)	8.08 (5.89)	−4.16 (6.60)	17.19 (6.49)	4.95 (6.73)	−9.11 (8.50)	0.290
Constipation	14.84 (4.83)	13.63 (5.17)	−1.21 (4.02)	3.64 (5.57)	−11.19 (4.11)	9.99 (5.49)	0.077
Diarrhoea	8.89 (3.64)	9.03 (3.77)	0.14 (3.32)	3.76 (4.15)	−5.13 (3.39)	5.27 (4.38)	0.237
Financial Difficulties	15.08 (4.76)	21.64 (6.72)	6.56 (4.54)	20.49 (6.76)	5.41 (4.65)	1.15 (6.49)	0.861
**MMRC Dyspnea Scale**	1.03 (0.17)	0.99 (0.19)	−0.04 (0.15)	0.96 (0.20)	−0.07 (0.15)	0.03 (0.20)	0.889
**PHQ-9 Total Score (omits Q10)**	3.55 (0.62)	2.72 (0.70)	−0.83 (0.65)	3.86 (0.78)	0.31 (0.68)	−1.14 (0.88)	0.203

Notes

EORTC Quality of life (QoL) score and functioning domains: higher score = better QoL/functioning.

EORTC Symptom Domains: higher score = worse symptoms.

MMRC Dyspnea Scale: higher score = worse dyspnea.

PHQ-9: higher score = worse depressive symptoms.

After controlling for baseline scores and sex, there was a significant between-group differences favoring the IG in the role functioning domain (17.04 (SE 7.16); *p* = 0.02) of the EORTC. The dyspnea scale of the EORTC neared statistical significance (− 13.31 (SE 6.62); *p* = 0.051). Four categories had improvement in the IG and worsening in the UC group, but between-group difference did not meet significance: PHQ-9 score (− 1.14 (SE 0.88); *p* = 0.20), fatigue (− 5.18 (SE 6.88); *p* = 0.46), nausea/vomiting (− 8.65 (SE 5.80); *p* = 0.14), appetite loss (− 9.11 (SE 8.50); *p* = 0.29). Three categories had worsening in the IG and improvement in the UC group, but between-group difference did not meet statistical significance: EORTC social functioning domain (− 8.62 (SE 7.45); *p* = 0.25), insomnia (4.07 (SE 8.71); *p* = 0.64), and diarrhea (5.27 (SE 4.38); *p* = 0.24). Both groups reported improvements in several categories without between-group significance: MMRC dyspnea scale (0.03 (SE 0.20); *p* = 0.89), EORTC physical functioning domain (− 0.64 (SE 3.41); *p* = 0.85), EORTC emotional function domain (− 0.43 (SE 3.83); *p* = 0.91), EORTC cognitive function domain (− 2.05 (SE 3.13); *p* = 0.52), and constipation (9.99 (SE 5.49); *p* = 0.08). Both groups reported worsening without between-group difference in their EORTC pain (0.41 (SE 6.28); *p* = 0.95) and financial difficulty (1.15 (SE 6.49); *p* = 0.86) scores.

In the IG, increases in step count were positively correlated with improvements in multiple aspects of the EORTC, including the QLQ summary score, role functioning domain, emotional functioning domain, nausea/vomiting, and constipation. In both groups, increase in self-reported PA only correlated with improvement in the role functioning domain of the EORTC (Table 5).

**Table 5:** Pearson Correlation Between Physical Activity and Survey Score Changes

	Change in Exercise Minutes (n = 40)		Change in Step Counts (***n*** = 20)	
Score Type	Pearson Correlation	***p***-value	Pearson Correlation	***P***-value
EORTC QLQ-C30				
QLQ summary score	0.19	0.261	**0.54**	**0.021**
Global health status / QoL	0.03	0.865	−0.06	0.813
Physical functioning	0.09	0.589	0.24	0.344
Role functioning	**0.32**	**0.044**	**0.60**	**0.007**
Emotional functioning	0.08	0.638	**0.54**	**0.016**
Cognitive functioning	0.20	0.226	0.43	0.067
Social functioning	−0.11	0.504	0.29	0.232
Fatigue	−0.24	0.142	−0.42	0.074
Nausea and vomiting	−0.12	0.457	**−0.46**	**0.046**
Pain	0.03	0.846	−0.20	0.409
Dyspnoea	−0.22	0.172	−0.16	0.521
Insomnia	−0.08	0.616	−0.24	0.332
Appetite loss	−0.13	0.439	0.22	0.365
Constipation	0.10	0.547	**−0.54**	**0.017**
Diarrhoea	0.01	0.954	0.26	0.279
Financial difficulties	0.10	0.562	−0.08	0.758
MMRC Dyspnea Scale: Description of breathlessness	−0.07	0.683	−0.15	0.568
PHQ-9 Total Score (omits Q10)	−0.21	0.220	−0.01	0.962

*Footnote: Quality of life and symptom questionnaires were collected at week 12. Readers should note that the Intervention Group (n = 20) had increased walking distance at week 6 (but not at week 12; see Table 2)

### Biomarkers

Only 10 subjects (25%) provided both baseline and 3-month fasting blood samples (IG *n* = 6; UC *n* = 4). There were no significant differences in insulin, leptin, CRP, or PD-L1 (Table 6). Only sPD-1 showed significant between-group change compared to baseline (219.79 (SD 54.47); *p* < 0.001), with increase in the IG and decrease in UC.

**Table 6:** Test of Biomarker Change Using a Linear Mixed Model^a^

		Intervention Group		Usual Care			
Biomarker	Week 1	Week 12	Change	Week 12	Change	Difference of change	***P-*** value
Insulin (uIU/mL; SD^b^)	16.81 (2.53)	21.89 (7.50)	5.08 (7.29)	25.12 (7.31)	8.32 (7.01)	−3.24 (10.11)	0.752
Leptin (ng/mL)	21.84 (5.63)	22.18 (5.08)	0.34 (3.28)	18.53 (5.84)	− 3.31 (3.50)	3.65 (4.55)	0.430
C-Reactive Protein (CRP; ng/mL)	4344.41 (459.07)	2963.07 (650.29)	− 1381.34 (682.02)	4205.55 (717.93)	−138.87 (665.26)	− 1242.47 (918.90)	0.188
Soluble PD-1 (pg/mL)	**90.51 (19.86)**	**243.27 (36.44)**	**152.76 (45.51)**	**23.48 (38.69)**	**−67.03 (41.49)**	**219.79 (54.47)**	**< 0.001**
Soluble PD-L1 (pg/mL)	9.76 (0.34)	10.98 (8.14)	1.22 (8.18)	22.06 (7.64)	12.30 (7.64)	−11.08 (11.17)	0.331

^a^ Only *n* = 10 patients provided baseline and follow-up samples; 6 in the intervention group and 4 in usual care

^b^ SD = Standard Deviation

### Adverse events

There were 4 serious adverse events unrelated to the study but occurring during the study period, including 3 hospitalizations (chronic obstructive pulmonary disease exacerbation, pneumonia, and hyperthyroidism) and 1 ER visit (fall). There were 2 reported minor adverse events (ankle pain and bronchitis) that were also unrelated to the study. Relationship to the study was determined by discussion with the patients (minor events) and their clinicians (major events).

### Patient satisfaction

Satisfaction and feedback were evaluated via “yes/no” responses. In the intervention group, 17/20 (85%) patients reported feeling that the intervention was helpful, 18/20 (90%) would participate in a future activity study, 19/20 (95%) plan to continue walking for exercise, and 17/20 (85%) plan to continue tracking their activity.

## Discussion

The goal of our PA trial was to determine interest in participating in our 3-month home-based walking regimen using wrist-bound accelerometers, individualized walking goals, and daily text messaging in patients with advanced stage lung cancer. Among patients screened for eligibility, 12% were excluded by their clinician and 17% of eligible patients were not interested. We enrolled 56% of eligible patients. As evidenced by infrequent provider recommendations, patients with lung cancer (especially stage III or IV NSCLC) are often not considered for PA interventions [45, 46]. Yet, our study showed high interest in participation and satisfaction with the exercise program. Since most patients with lung cancer are diagnosed with advanced stage disease, [1] ensuring their safety, eligibility, and interest in PA regimens is critically important. Our study confirms that, in contrast to conventional wisdom, many patients with advanced NSCLC are interested and able to participate in home-based PA interventions.

Our home-based intervention improved PA in patients with advanced NSCLC. Interestingly, both groups increased their self-reported PA (Table 2), though the IG increased their PA more than the UC group. Since subjects were participating in a PA intervention, it is logical that both groups would attempt to increase their activity. The IG reported increasing their weekly PA by an average of 123 min. Though discrepancies between self-reported and monitored PA levels are recognized, [27] an increase of PA greater than 2 h/week is clinically significant. Step count data in the IG suggested improvement during the first 6 weeks, though there was no statistically significant change during the study. Discordance between self-reported PA and step counts may be multi-factorial, with patients increasing their PA soon after receiving their FitBit®, potentially not wearing their FitBit® during all walking sessions, or performing other types of PA (e.g., weights or cycling). Though wearable devices increase PA, [47, 48] both PA and device use may wane with time [49, 50]. For example, 1/3 of consumers report they stop using the device within 6 months [49]. Wang and colleagues’ also showed increased PA predominately during the first portion of the intervention [51]. More work is needed to determine if patients’ device adherence wanes with time or their PA takes other forms not recognized by step counting. Our findings suggest that timing of home-based PA interventions may be important, since results may be the most effective early after the intervention.

Adherence to the study’s PA recommendations (i.e., average step count + increments of 400 steps/day) was lower than expected. Since our primary goal was to **slowly and safely** increase patients’ PA levels at home, we recommended a mild increase in patients’ baseline step count, which has been safe and effective in other studies [43, 52]. Longer walking distance [53] or percentage increases of baseline walking distance have also been utilized [54]. Since the choice of step count goal is variable and our study shows potential benefit despite low adherence, we interpret our findings that **any increase in PA** has potential clinical benefit. This suggestion is supported by Thompson and Eijsvogels’ editorial to “The Physical Activity Guidelines for Americans,” wherein they highlight that the most benefit is obtained from transitioning from inactivity to “even small amounts of, physical activity …” [55, 56].

Patients receiving the intervention reported a clinically-significant significant improvement in the role functioning domain of their QoL, and dyspnea scores trended toward statistical significance. Role functioning references patients’ limitations in their work, daily activities, hobbies, and leisure time activities. This study, therefore, suggests that our home-based intervention allows patients to maintain their lifestyle and independence. We highlight two points. First, the UC group in this study reported significant *worsening* in the role functioning domain of the EORTC, resulting in a between-group difference of 17. The EORTC defines a change of 10–20 as “moderate,” [31, 36] and we interpret a ~ 20% change in role functioning as likely clinically significant. Since many patients with lung cancer experience worsening in their QoL and symptoms with time, [2] our study supports the notion that PA interventions may prevent functional loss or symptom worsening that occurs during lung cancer treatment. Since improvement in the IG (and worsening in the UC group) was also noted in the global health status and multiple symptoms, a larger study may identify even more clinical benefit. Indeed, due to our study’s small size and multiple QoL domains that did not reach statistical significance, our study cannot be considered definitive, and a larger trial is needed. In addition, since walking-based PA was the highest at ~week 6, more frequent questionnaires in future studies may clarify the trend of patients’ QoL. Second, among the IG, strong correlations with higher QoL and lower symptom burden were noted in patients who increased their week-to-week activity but did not necessarily reach the recommended study goal. It is important to note that 3 categories had non-statistically significant worsening in the IG but improvement in the UC group: social functioning (i.e., family life and social activities), insomnia, and diarrhea. Though these topics deserve more study in future work, it seems less likely that these symptoms would be worsened by our intervention. To summarize, our findings suggest that *any* physical activity has the potential to improve QoL and symptoms in lung cancer survivors with advanced stage disease.

For context, it is worth noting that the EORTC recently published Thresholds of Clinical Importance (TCIs) for each of the functional and symptom domains in the EORTC QLQ-C30 [57]. The TCIs were established to identify clinical significance of single value (in contrast to changes with time). Clinical significance relied on limitations in daily living, perceived need for help/treatment, or associated worry. Regarding role functioning (which was clinically and statistically significant in this study), our cohort had a higher baseline score (86) than the TCI (58). Several aspects make this comparison of unclear significance to our findings. First, our study did rely on change of EORTC with time (rather than a static value). Second, the TCI relied on multiple cancer types, and lung cancer comprised only ~ 10% of this cohort. Third, clinically significant impairments in role functioning may reflect impairments in activities of daily living (ADLs), which are likely less common in patients participating in a PA-focused clinical trial.

The preliminary finding of sPD-1 increasing in the IG is hypothesis-generating. Though the clinical significance of soluble PD-1 and PD-L1 is currently unknown, [58] increasing PA may bolster the anti-tumor immune response, potentially via modulating PD-1. Since our sample size was limited, a larger study would be needed to prove this hypothesis. The low portion of patients providing both blood samples is attributed to (1) many patients not wanting an additional blood draw since they were undergoing regular phlebotomy during their systemic cancer treatment and (2) the coronavirus-19 pandemic impairing all of our sample acquisition at the latter portion of the study.

Our study has several limitations. First, as a pilot study, the sample size was small, and our findings need validation in a larger cohort. The finding of potential benefit despite our small sample size is encouraging. Second, there was a higher proportion of female patients in UC group. It is possible that sex may contribute to an individual’s likelihood to participate in a PA trial utilizing mobile health (mHealth) technology. Third, the true participation rate for our intervention is unknown. Since 27% of patients undergoing chart review were not able to be approached during the study period and the most exclusions were for not meeting study criteria, the true applicability of our intervention to lung cancer survivors is likely higher than 56%. A longer study period and larger enrollment target would have overcome scheduling challenges and pharmacologic trial competition. Fourth, our study provided a combined intervention, including education, a wrist-bound accelerometer, text messaging, and (for some patients) phone calls to evaluate for technical difficulties; it is unclear from this analysis which component(s) led to a change in behavior and/or QoL. Finally, we were not able to confirm whether text messages were received or read by subjects. An automated system (rather than our individual delivery of texts) would be preferable for future, larger interventions.

Two findings in our study suggest the direction PA interventions for lung cancer patients should take in the future. First, the notion that any intervention that safely increases PA will improve clinical outcomes should motivate clinicians to “target” PA regimens to individual cancer patients. The considerable number of patients excluded due to “inability to safely walk” or “no access to a smartphone” could be overcome by utilizing supervised regimens (i.e., pulmonary rehabilitation) or provision of smartphones, respectively. Such an individualized approach would likely optimize participation, adherence, and clinical benefit. Second, future research utilizing mHealth should utilize automated messaging. Importantly, though a limitation due to our study’s texting delivery system, mHealth interventions hold incredible potential to expand the delivery of PA interventions to lung cancer survivors.

## Conclusions

In summary, our pilot study found that a home-based PA intervention using mHealth may be applicable to over half of eligible patients with stage III/IV NSCLC. Our study also suggests that the intervention increases PA,is safe, and has potential clinical benefit with regard to symptoms and QoL. To our knowledge, this is the first randomized study utilizing mHealth to deliver home-based PA and evaluate QoL in patients with advanced stage NSCLC. In the setting of global viral pandemic, home-based PA interventions hold enormous potential to improve (or maintain) QoL during lung cancer survivorship. Future studies should utilize home-based PA in all-stage lung cancer survivors, enroll larger cohorts, confirm predictors of participation, and compare results to other PA interventions. The results of these studies will facilitate “targeting” effective PA regimens to patients who are most likely to benefit.

## Data Availability

The datasets used and/or analysed during the current study are available from the corresponding author on reasonable request. All authors had full access to the de-identified data. BCB maintained and protected the identified data. As a study funded, in part, by the NIH, de-identified data will be uploaded to clinicaltrials.gov.

## Abbreviations

QoL: Quality of life
PA: Physical activity
IG: Intervention group
UC: Usual care
NSCLC: Non-small cell lung cancer
sPD-1: Soluble programmed cell death protein-1
sPD-L1: Soluble programmed cell death protein ligand-1
CRP: C-reactive protein
EORTC-QLQ-C30: European Organisation for the Research and Treatment of Cancer Quality of Life Core Questionnaire
PHQ-9: Patient Health Questionnaire-9 depression scale
SE: Standard error
