# Correction: Randomized trial of physical activity on quality of life and lung cancer biomarkers in patients with advanced stage lung cancer: a pilot study

**DOI:** 10.1186/s12885-024-13183-9

**Published:** 2024-11-15

**Authors:** Brett C. Bade, Geliang Gan, Fangyong Li, Lingeng Lu, Lynn Tanoue, Gerard A. Silvestri, Melinda L. Irwin

**Affiliations:** 1 Northwell Health, New Hyde Park, NY USA; 2 Yale Center for Analytical Sciences, Yale School of Public Health, New Haven, USA; 3 Department of Chronic Disease Epidemiology, Yale University School of Public Health, New Haven, USA; 4 Department of Internal Medicine, Section of Pulmonary, Critical Care, and Sleep Medicine, Yale University School of Medicine, P.O. Box 208057 300 Cedar Street TAC – 441 South, New Haven, CT 06520-8057 USA; 5 Division of Pulmonary, Critical Care, and Sleep Medicine, Department of Internal Medicine, Medical University of South Carolina, New Haven, USA

**Correction: BMC Cancer 21, 352 (2021)**

10.1186/s12885-021-08084-0

Following publication of the original article [1], the authors report an error. Table 3 (Baseline Patient-Reported Outcomes by Study Group) incorrectly references standard error (SE). In fact, the parenthetical numbers represent standard deviation (SD). Standard error (SE) is correctly referenced in Table 4 (Overall Patient-Reported Outcomes by Study Group (Mixed Effects Model)). The findings and Conclusions of the paper are unchanged.

The authors apologize for the oversight.
